# Transvaginal Sonographic Features of Diffuse Adenomyosis and Associated Symptoms in 18–30-Year-Old Nulligravid Women Without Endometriosis: Is Our Diagnosis Still Valid 10 Years Later? A Re-Evaluation of Previous Results

**DOI:** 10.3390/jcm15145554

**Published:** 2026-07-15

**Authors:** Irene Colombi, Francesco Giuseppe Martire, Eugenia Costantini, Ilaria Ianes, Luca Labanca, Caterina Exacoustos, Errico Zupi, Lucia Lazzeri

**Affiliations:** 1 Gynecology and Obstetric Department, Azienda USL Toscana Centro, Santo Stefano Hospital, 59100 Prato, Italy; colombi.irene1@gmail.com (I.C.); zupi@unisi.it (E.Z.); 2 Department of Molecular and Developmental Medicine, Obstetrics and Gynecological Clinic, University of Siena, 53100 Siena, Italy; eugenia.costantini22@gmail.com (E.C.); ilaria.ianes@gmail.com (I.I.); lucalabanca@gmail.com (L.L.); lucialazzeri79@gmail.com (L.L.); 3 Department of Surgical Sciences, Obstetrics and Gynecological Clinic, University of Rome Tor Vergata, Viale Oxford, 81, 00133 Rome, Italy; caterinaexacoustos@tiscali.it

**Keywords:** adenomyosis, ultrasound, MUSA criteria

## Abstract

**Background:** The introduction of the Morphological Uterus Sonographic Assessment (MUSA) 2022 criteria for diagnosing adenomyosis has reduced diagnoses compared with previous criteria. However, the impact of each ultrasonographic sign on diagnosis and symptom management remains unexplored. **Methods:** We retrospectively re-evaluated data from our 2015 study, which used criteria available before the first MUSA consensus. Nulligravid women aged 18–30 years without endometriosis were included to determine whether MUSA 2022 criteria result in fewer diagnoses than previous criteria. Sonographic images were reviewed and MUSA 2022 criteria applied. The primary outcome was to estimate previous overdiagnosis; the secondary outcome was to identify correlations between ultrasound features and symptoms. **Results:** Among 156 patients, at least one direct MUSA 2022 sign was present in 36 women; 17 had only indirect signs; and 103 had no ultrasound signs. MUSA 2022 criteria reduced adenomyosis prevalence from 34% to 23.1%. Direct signs were associated with greater dyspareunia (44.4% vs. 11.8%) and higher VAS scores. Hypermenorrhoea was associated with asymmetrical thickness (Odds Ratio 2.84) and a globular uterus (Odds Ratio 4.03). **Conclusions:** Applying MUSA 2022 criteria reduces adenomyosis diagnoses, suggesting that previous criteria may have overestimated prevalence. Nevertheless, ultrasound remains an adjunct to clinical assessment, and symptoms should guide management when direct signs are absent.

## 1. Introduction

Adenomyosis is a benign gynecological condition characterized by the infiltration of endometrial glands and stroma within the myometrium, generally associated with surrounding hyperplastic smooth muscle fibres.

Symptoms are variable and patients may be completely asymptomatic or present with chronic pelvic pain, abnormal uterine bleeding and reproductive dysfunction [1].

The prevalence of adenomyosis is complex to estimate for several reasons. The first bias is that adenomyosis and endometriosis are often co-morbid, so prevalence estimates for either condition alone are not accurate. In addition, the diagnosis has historically been histological, but hysterectomy is a procedure generally reserved for older or symptomatic women. Since we know that adenomyosis can also affect adolescents [2] or asymptomatic women, it is clear that the histological approach to diagnosis may lead to an underestimation of the prevalence of the disease.

Histological diagnosis is also no longer required, the use of non-invasive techniques including magnetic resonance (MRI) and transvaginal ultrasound (TVS) are considered valid diagnostic tools. There are no statistically significant differences in the diagnostic accuracy of the two methods (MRI sensitivity of 78% and specificity of 88% vs. 2D-TVS 74% and 76%, and 3D-TVS 84% and 84%), with the advantage of TVS being more accessible, immediate and less expensive [3]. However, prevalence estimates vary depending on the method employed, and standardization has become increasingly necessary over time. Standardized ultrasound criteria may also be useful for the early identification of diffuse adenomyosis, particularly in adolescents and young women, in whom histological confirmation is rarely feasible [4,5,6].

In 2015, we published a paper [7] that aimed to identify ultrasound signs of adenomyosis in a cohort of 18- to 30-year-old nulligravid women without endometriosis who had not been exposed to uterine procedures, surgery or disease, and to examine the association of these features with symptoms of dysmenorrhea and abnormal uterine bleeding [7]. The ultrasound criteria used to diagnose adenomyosis were: (1) heterogeneous myometrium, described as an irregular echogenic structure of the myometrium with decreased or increased echogenicity; (2) hypoechogenic striations in the myometrium causing parallel shadowing; (3) anechoic myometrial rounded areas forming lacunae or cysts; (4) asymmetric myometrial thickening of the uterine wall. The diagnosis of adenomyosis was made in the presence of at least one of these signs and was supported by the results of two of our previous publications [8,9]. Applying these criteria, the study showed a prevalence of adenomyosis of 34% in the study population. Asymmetry of uterine wall thickness was the most common sign observed. The analysis also showed an association between the presence of ultrasound signs of adenomyosis and dysmenorrhea (*p* = 0.005) and a high Pictorial Blood Loss Assessment Chart (PBAC) score for menstrual loss (*p* = 0.03).

At the time, however, the literature was not uniform due to the lack of a common standard for the ultrasound diagnosis of adenomyosis.

The first attempt to standardize the sonographic diagnosis was made by the Morphological Uterus Sonographic Assessment (MUSA) [10] in 2015. The proposed diagnostic features follow the criteria used for diagnosis back then, which were also adopted in our study, but provide greater standardization. These criteria include asymmetrical thickening of uterine walls, cysts, hyperechoic islands, fan-shaped shadowing, echogenic subendometrial lines and buds, translesional vascularity, irregular junctional zone and interrupted junctional zone.

Now, more than 10 years later, we can rely on new advances in ultrasound diagnosis, as in 2022 the MUSA criteria were updated [6] through a Delphi procedure to revise the previously proposed definitions. The main differences include the distinction between direct and indirect features of adenomyosis. The direct signs are: myometrial cysts of any size, hyperechoic islands appearing as hyperechogenic areas within the myometrium unconnected to the endometrium, and subendometrial lines and buds disrupting the junctional zone and penetrating perpendicularly into the myometrium, which are direct sonographic signs of the presence of ectopic endometrium within the myometrium. Indirect signs, on the other hand, are features that occur as a result of presence of ectopic endometrium and appear as muscular hypertrophy or artefacts. These signs are: the globular appearance of the uterus, asymmetry of wall thickness, fan-shaped shadowing, increased translesional vascularity, irregularity of the junctional zone or its disruption. According to MUSA 2022 [6], the diagnosis can only be made if direct signs are present; if only indirect signs are observed, the diagnosis remains uncertain.

Nevertheless, recently an important role of indirect signs has also been suggested [11] since experienced sonographers often diagnose adenomyosis on the basis of thickened hypertrophic myometrial fibrotic reaction, globular uterus and on JZ irregularities without direct signs.

**Figure 1:**
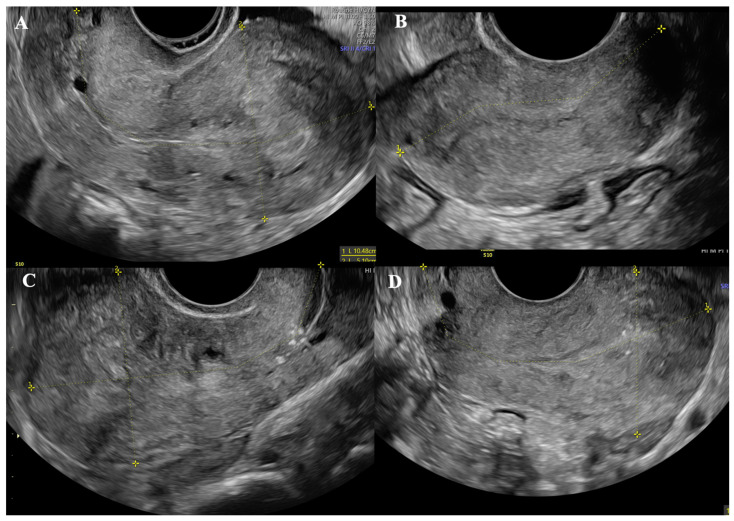
Examples of the main sonographic features of adenomyosis. Myometrial cysts (**A**), hyperechoic islands (**A**), subendometrial lines and buds (**D**), globular appearance of the uterus (**A**–**D**), asymmetry of wall thickness (**A**–**C**), fan-shaped shadowing (**A**).

Examples of different sonographic features of adenomyosis are shown in Figure 1.

The different diagnostic methods and criteria have led to different results in terms of correlation with symptoms and reproductive outcome. The need to standardize the diagnosis of adenomyosis to correlate research studies is highly requested, since the diagnosis made in older studies seems not comparable to those published in recent years. The need to clarify the role of individual ultrasound criteria in diagnosing both the presence and absence of adenomyosis is of considerable clinical relevance. Nevertheless, the diagnostic significance of these criteria may extend beyond their simple identification. Indeed, the type, localization, distribution, and extent of adenomyotic lesions appear to influence not only disease expression and symptom burden but also reproductive outcomes. A deeper understanding of how these ultrasound features correlate with clinical and reproductive parameters may improve disease characterization and support a more personalized approach to patient management. With a new standard for diagnostics, it was our duty to re-evaluate our previous work.

The aim of the present study was to re-evaluate the diagnosis of adenomyosis made in 2013 in our previous cohort of patients (Pinzauti et al. published in 2015 [7]) using the MUSA 2022 diagnostic criteria, in order to identify possible discrepancies between the 2013 and 2025 diagnoses and to estimate the likelihood of reduction in diagnosis after reclassification. Secondly, we wanted to identify a possible correlation between the ultrasound features and the reported symptoms, also by comparing these data with those from the literature.

## 2. Materials and Methods

This was a retrospective study with the purpose of re-evaluating data from our previous study [7] published in 2015 on the ultrasound diagnosis of adenomyosis considering the updated direct criteria proposed by the 2022 MUSA consensus.

Additional ethics approval was not required, as the original study and data management had already been approved by the Institutional Review Board of the University of Siena in 2015. The present study was limited to a retrospective re-analysis of previously collected and anonymized ultrasound images and clinical data, with no new patient recruitment, intervention, patient contact, or additional data collection.

In the previous study, patients were enrolled between April 2013 and July 2013, they were nulligravid women, between 18 and 30 years old, with contraception request, who underwent a transvaginal ultrasound assessment at the gynecological outpatient clinics of Le Scotte Hospital in Siena. Women were excluded if they had a past or present history of endometriosis, fibroids, ovarian cysts, endometrial pathology, current use of hormonal therapies or medications that would interfere with the menstrual cycle, previous uterine surgery, or a history of infertility. The amount of menstrual blood loss was subjectively assessed using a pictorial blood loss analysis chart (PBAC), and heavy/abnormal bleeding (menorrhagia) was defined as a PBAC score ≥ 100. Pain severity was assessed using a visual analogue scale (VAS) from 0 to 10, with 0 indicating no pain and 10 indicating intolerable pain. All data were accurately recorded in an anonymized database.

All patients underwent a detailed clinical assessment followed by 2D and 3D TVS by an experienced sonographer and all myometrial characteristics were reported. In the 2013 study, in accordance with previous papers [12,13], the 2D-TVS diagnostic features for adenomyosis were: heterogeneous myometrium, hypoechoic striation in the myometrium, myometrial anechoic cysts, asymmetrical myometrial thickening of the uterine walls. A 3D-TVS evaluation was considered suggestive of adenomyosis if JZmax ≥ 8 mm and/or JZdiff ≥ 4 mm. The evaluation was considered suggestive for adenomyosis when at least one of these recognized features was observed.

For the present study, data from the previously processed database were retrieved and re-analyzed in the context of the MUSA 2022 criteria. In addition, all stored ultrasound images were retrospectively reviewed by a single experienced sonographer with over 10 years of expertise in the diagnosis and ultrasound characterization of adenomyosis, ensuring a consistent reassessment and classification of all sonographic features according to the updated MUSA 2022 framework.

In order to correlate the ultrasound features observed in 2013 with the MUSA 2022 diagnostic criteria, we considered as direct signs the presence of intra-myometrial cysts and of hyperechogenic islands. Although lines and buds were not included among the sonographic features assessed in the original 2013 study, they were specifically evaluated in the present analysis and classified as direct signs. Indirect signs were considered to be asymmetry of uterine wall thickness, globular appearance of the uterus, presence of fan-shaped shadowing within the myometrium and increased myometrial vascularity.

**Figure 2:**
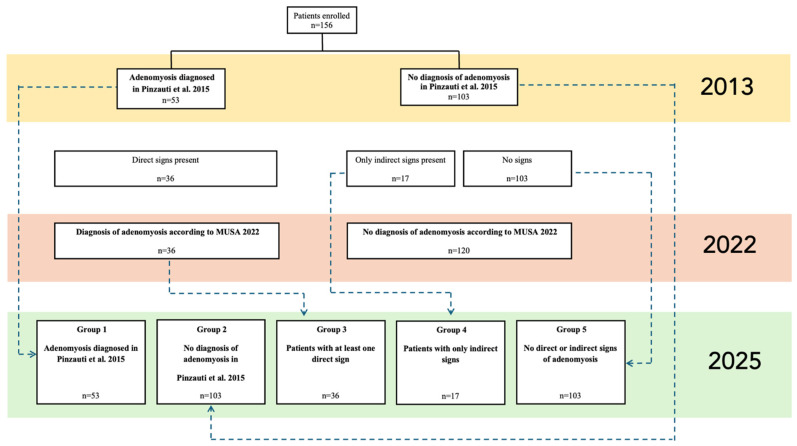
Diagram showing the subdivision into patients with and without adenomyosis according to the criteria applied at the time of data collection in 2013 (in yellow); subdivision into patients with and without adenomyosis according to the MUSA 2022 criteria (in orange); and the groups formed in the present study with the re-evaluation of the database carried out in 2025 (in green) [7].

A total of 156 patients were enrolled. For the purpose of our investigation, we created five study groups. Group 1 consisted of women who had been diagnosed with adenomyosis in the paper by Pinzauti et al. (*n* = 53), while group 2 consisted of those who had not been diagnosed (*n* = 103). Re-analysis of the images by subdividing the patients according to the presence or absence of at least one direct sign of adenomyosis resulted in 3 further groups. Group 3 consisted of patients with at least 1 direct sign of adenomyosis (*n* = 36), group 4 included patients with only indirect signs of adenomyosis (*n* = 17), and group 5 included women with no direct or indirect signs of adenomyosis (*n* = 103). The construction process of the groups compared in this study is shown in Figure 2.

Graph Pad Prism 5 (San Diego, CA, USA) was used for statistical analysis. There were no missing data. Patient characteristics were analyzed using univariate statistical analyses to define the study population. Pearson’s χ^2^ test and Fisher’s exact test were used to compare qualitative variables (categorical data), while Student’s *t*-test and ANOVA with post hoc test were used to evaluate quantitative data. Results are presented as mean ± SD or %. *p* < 0.05 was considered statistically significant.

## 3. Results

The results of the previous study reported 53 cases of adenomyosis diagnosed according to the diagnostic criteria used at the time of data collection. The remaining 103 women had not been diagnosed with adenomyosis by the sonographer. A re-evaluation of the same cohort of patients in the current study showed the presence of at least one direct sign according to the MUSA 2022 criteria in 36 women, the presence of only indirect signs in 17 women, and no signs of adenomyosis, either direct or indirect, in 103 women. Patient characteristics, symptoms and menstrual cycle pattern are reported in Table 1.

The prevalence of adenomyosis calculated in the previous paper was 34%; using the MUSA 2022 criteria, the prevalence was found to be 23.1%, with a reduction in the diagnosis rate of 10.9%.

The data on the presence of direct signs are identical to those previously published, as they come from the same database. Following image re-evaluation, echogenic subendometrial lines and buds were identified in 14 patients, corresponding to 38.9% of women with adenomyosis and 9.0% of the overall study population. The simultaneous presence of direct and indirect signs was observed in 69.44% of cases. In particular, there was asymmetry of wall thickness in 18 patients and fan-shaped shadowing in 7 cases.

In the group of patients with only indirect signs, i.e., group 4 (*n* = 17), asymmetry of wall thickness was present in all cases (100%), while fan-shaped showing were described in 41.18% of women and increased myometrial vascularization in 70.59%. The combination of two or more indirect signs was present in 70.59% of cases.

Post hoc analysis with a Tukey test was performed on significant data. Tukey test analyses showed a significant difference in the duration of menstruation between groups, with Group 3 having a shorter duration (4.80 ± 0.87 days) than the other groups (*p* = 0.001).

Tukey’s analysis also showed a significant difference in menstrual bleeding (PBAC score) between Group 3 and Group 4, with Group 3 having more intense bleeding (*p* = 0.003). In Group 5 PBAC score significantly higher than Group 4 (*p* = 0.0005).

## 4. Discussion

In our previous study [7], adenomyosis was diagnosed in 53 out of 156 women, corresponding to a prevalence of 34%. In the present study, after re-evaluating the data according to the MUSA 2022 consensus, 36 patients were diagnosed, corresponding to a prevalence of 23.1%. This represents an absolute decrease in prevalence of 10.9 percentage points (from 34% to 23.1%) when only direct signs of adenomyosis were considered.

### 4.1. Direct Signs

In regard to direct signs, authors agree that myometrial cysts are the most specific sign [8,14] while hyperechogenic islands are the most sensitive [8]. In a cohort of women aged 28–54 years with histological confirmation of adenomyosis, Krentel et al. [11] found that the best preoperative ultrasound sign predictive of pathology was the presence of subendometrial microcysts. This feature showed a diagnostic accuracy of 56.7% and its detection increased the probability of anatomopathological confirmation of the pathology threefold.

Other authors state that lines and buds are the best individual diagnostic criterion [12,15]. While this feature was not assessed in the original study, re-evaluation of the stored ultrasound images according to the MUSA 2022 criteria allowed its identification in 14 patients with adenomyosis (38.9%). This prevalence is consistent with that reported in recent studies adopting the revised MUSA classification, in which echogenic subendometrial lines and buds were observed in approximately 46% of women with adenomyosis. Furthermore, recent validation studies have identified this feature as one of the most predictive individual sonographic markers of adenomyosis [16].

### 4.2. Indirect Signs

In our previous work, the diagnosis of adenomyosis was also made in the presence of only indirect signs (32%). The role of these sonographic features has changed over time and is still debated, although they are not considered diagnostic, we cannot ignore their association with pathology. In previous papers analyzing the presence of indirect signs [11], in fact, the detection of hypoechoic striae showed a specificity of 85.9% but a low sensitivity of only 28.7%. The most predictive indirect sign of adenomyosis is a globular uterus (*p* = 0.02), according to Yavuz et al. [12].

The asymmetry of uterine wall thickness is associated with high false-positive and false-negative rates and may be influenced by transient myometrial contractions, which are a recognized source of diagnostic error in uterine imaging [17]. In our cohort, asymmetry of the uterine walls was the most common indirect sign. Our data confirm the high false-positive rate; however, it was consistently observed in all patients with myometrial cysts. This suggests that the presence of asymmetry should prompt a more careful search for direct signs of adenomyosis.

### 4.3. Number of Sonographic Features Required for Diagnosis

How many of the ultrasound features need to be present to diagnose the pathology is another open question in the literature. In the work of Krentel et al. [11], the combination of several ultrasound signs seems to improve prediction of disease compared to the presence of a single one. The combination of a globular uterus, wall asymmetry and myometrial heterogeneity was associated with a false positive rate of 29%. However, it is interesting to note that this value is reduced when the presence of subendometrial microcysts is observed in addition to these signs, in which case the false positive rate falls to 11.5%.

The globular uterus and asymmetry of the uterine walls therefore appear as ambiguous ultrasound signs. These features may be present in adenomyosis; however, in the absence of direct signs, a definite ultrasound diagnosis cannot be achieved.

Yavuz et al. [12] found that a number of features > 4 both direct and indirect is a valid cut-off for the diagnosis of adenomyosis (*p* < 0.001), with the association of 3 direct and 2 indirect signs as the best with a diagnostic accuracy of 80%, positive predictive value of 100 and negative predictive value of 95.

According to Haj Hamound et al. [1], women with histological confirmation of adenomyosis had three or more concurrent features on ultrasound. In contrast, women without histological confirmation of the disease had fewer than two features. These data are confirmed by other authors, for example, Rasmussen et al. [18] found that two or more 2D/3D findings provided a more objective diagnosis. However, none of the studies cited distinguished between direct and indirect signs.

### 4.4. Correlation Between Ultrasound Features and Symptoms

It has been hypothesized that observing the number of features present may not only improve diagnostic accuracy but may also correlate with clinical presentation.

Naftalin et al. [19], have shown, in agreement with other literature data [13,20] that a higher number of ultrasound signs correlates with a higher pain score. In addition, a higher number of ultrasound features is associated with a higher PBAC [21], more severe symptoms, especially heavy menstrual bleeding (HMB), chronic pelvic pain (CPP) with VAS > 7 and dysmenorrhea with VAS > 7 [22].

Although the literature tends to agree that a high number of ultrasound signs may correlate with a worse symptomatic presentation, it is also possible that small, isolated lesions may be extremely symptomatic, whereas diffuse pathology may show mild symptoms [23].

In the light of the above, the spontaneous question is whether there is a correlation between a particular ultrasound sign and a symptom.

In the literature, the authors found that dysmenorrhea was more strongly associated with the presence of direct signs (OR 1.13) [22]. These data are not confirmed in our study, as we found no statistically significant differences in the presence of dysmenorrhea between women with direct signs of adenomyosis and those with only indirect signs (83.33% vs. 70.59%, *p* = 0.290), nor in the VAS. On the other hand, our study shows that the presence of direct signs of adenomyosis is associated with a higher occurrence of dyspareunia (44.44% vs. 11.76% *p* = 0.02) and a higher VAS than that reported by women with only indirect signs (*p* = 0.05).

Conversely, HMB appears to be associated with myometrial thickness asymmetry (OR 2.84), interrupted junctional zone (OR 2.02) and globose uterus (OR 4.03) [22]. Therefore, heavy menstrual bleeding seems to have a higher correlation with the presence of indirect signs of adenomyosis.

Our data showed a significant difference in menstrual cycle duration between the groups. In the presence of direct signs of adenomyosis, we observed a shorter menstrual duration compared to the other groups, but a higher PBAC score, suggesting that in the presence of direct signs, cycles are more likely to be shorter, but menorrhagia is more frequent. Moreover, in our cohort, women with no direct signs of adenomyosis showed a longer mean cycle duration (5.79 days) than those with direct features (4.80 days) (*p* = 0.001). Heavy menstrual bleeding may be due to a number of factors, including increased uterine volume and vascularization, altered contractility, and changes in the hormonal milieu with elevated estrogens and prostaglandins [24]. This condition can be related to adenomyosis, but it is also possible that there are other underlying causes, which is why it is possible for some women to experience HMB even in the absence of any signs of adenomyosis.

A similar study to ours was carried out by Biasoli et al. [24]. In their work the most common ultrasound sign was the presence of hyperechogenic islands (66%), followed by intramyometrial cysts (40%), confirming what we have reported in our work. In their previous study, reclassification according to the MUSA 2022 criteria led to a 16% reduction in adenomyosis diagnoses, confirming the substantial impact of the revised criteria on lowering the prevalence of ultrasound-diagnosed adenomyosis.

It is essential to describe not only the presence of signs of disease, but also their location and extent in the myometrium. Different sites of disease appear to correlate with both possible pathogenetic origin and symptomatology [25]. With regard to the first aspect, the most widely accepted theories suggest that adenomyosis may result from two different mechanisms. One theory proposes that areas of the endometrium invade and grow into the myometrium by invagination through damaged areas at the junctional zone. This may cause lesions that are mainly in the inner myometrium. On the other hand, adenomyosis may originate in part from endometrial tissue at the level of the pelvis, with a mechanism in common with endometriosis. It can invade the uterine muscle from outside, creating a pattern of adenomyosis of the external myometrium [26].

A third theory is that isolated areas of adenomyosis not in contact with the endometrium or serosa may be derived from embryonic or adult stem cells undergoing metaplasia. Several attempts have been made in the literature to classify the extent of the disease [27], but to date there is no consensus. Given the different correlation of symptoms and obstetric outcomes of the different types of adenomyosis, it seems clear that a more precise distinction and description would be useful both for research purposes and in clinical practice. Accurate sonographic characterization may also help avoid diagnostic misclassification with other myometrial disorders, such as uterine fibroids, which may have consequences for treatment choice and follow-up [28].

Women with adenomyosis of the inner myometrium were more likely to have recurrent pregnancy loss (RPL) than those with adenomyosis of the outer myometrium, so differences in the location of adenomyosis are associated with different risks of RPL. In addition, outer myometrial adenomyosis was more frequently associated with the presence of concomitant endometriosis [29]. Bourdon et al. [30] also found that women with adenomyosis located in the outer myometrium were more likely to be younger and nulligravid and more likely to have concomitant endometriosis than women with adenomyosis located in the inner myometrium.

One of the strengths of the first version of our previous paper, and therefore of the present data review, is that the patient cohort is highly selected and consists of women without ultrasound evidence of endometriosis or previous surgery for endometriosis. This effectively eliminates the bias of overlapping symptoms between the two conditions. Whilst we are aware that adenomyosis and endometriosis are closely related, the opportunity to investigate only one of these conditions provides considerable insight into the impact of adenomyosis on its own. Other studies support this hypothesis; the myometrial aspect of patients with adenomyosis alone appears different from that of patients with both conditions; Matot et al. [31] found that direct signs were more common in women with adenomyosis alone. However, in order to assess the synergistic effect with endometriosis, further sample collections are required that take into account the co-existence of the conditions.

Furthermore, the study group is composed of nulliparous women of young age, thus eliminating the bias of potential onset of the disease following pregnancy, abortion, uterine interventions such as uterine cavity revisions, and moreover, they are not selected for infertility, as none of these patients had sought pregnancy.

A limitation of this study is certainly its retrospective nature and the re-evaluation of a pre-existing database. In addition, the retrospective review of ultrasound images was performed by a single experienced sonographer, precluding assessment of interobserver agreement and introducing the possibility of operator-dependent interpretation. Future studies should include independent assessment by multiple expert sonographers to improve diagnostic reliability.

Moreover, because of the retrospective design, not all possible non-adenomyosis-related contributors to pelvic pain or abnormal uterine bleeding could be assessed; therefore, the associations between sonographic features and clinical symptoms should be interpreted with caution.

We strongly believe that the presence of symptoms should guide the clinician. Our recommendation is that clinical management should be driven by symptoms and not solely by the presence or absence of direct sonographic signs. However, in asymptomatic cases, the absence of direct signs of disease may reduce overdiagnosis, which in turn may reduce the psychological impact on the patient as well as decreasing costs.

## 5. Conclusions

Accurate assessment of the patient with adenomyosis is of primary importance in order to ensure appropriate pre-conception counselling, pregnancy and delivery management and, for women who do not wish to have children, appropriate treatment according to the patient’s needs. By applying the MUSA 2022 criteria, the number of diagnoses of adenomyosis that would be made using the previous criteria is reduced. This has the effect of reducing potential overdiagnosis with significant psychological and economic benefits. However, ultrasound is an adjunct to the clinician; in the presence of symptoms, absence of direct ultrasound evidence should not be a criterion for non-treatment.

## Figures and Tables

**Table 1:** Patient characteristics, symptoms and menstrual cycle pattern. Data are presented as mean ± SD or *n* (%). * ANOVA. ^§^ Chi-square test. PBAC, pictorial blood loss analysis chart; VAS, visual analog scale.

	Group 1	Group 2	Group 3	Group 4	Group 5	*p* Value
	Adenomyosis Diagnosed in Pinzauti et al. 2015 [7]	No Diagnosis of Adenomyosis in Pinzauti et al. 2015 [7]	Patients with at Least One Direct Sign	Patients with Only Indirect Signs	No Direct or Indirect Signs of Adenomyosis	
	*n* = 53	*n* = 103	*n* = 36	*n* = 17	*n* = 103	
Age (Years)	26.06 ± 2.89	24.26 ± 3.27	26.33 ± 3.01	25.47 ± 2.15	23.71 ± 2.48	<0.00001 *
Height (cm)	167.21 ± 6.08	166.44 ± 6.69	167.33 ± 6.89	169.23 ± 4.58	166.36 ± 6.89	0.187 *
Weight (kg)	56.19 ± 5.73	58.24 ± 7.18	55.22 ± 4.52	58.52 ± 7.01	58.38 ± 7.72	0.386 *
Body mass index (kg/m^2^)	20.56 ± 1.95	21.39 ± 2.73	20.12 ± 1.55	20.84 ± 1.86	21.47 ± 2.86	0.870 *
Previous OC use	30.19%	19.42%	38.89%	41.18%	21.36%	0.084 ^§^
Regular menstrual cycle	79.25%	69.90%	63.89%	88.24%	66.02%	0.178 ^§^
Length of menstruation (days)	5.31 ± 1.41	5.58 ± 1.11	4.80 ± 0.87	5.05 ± 1.43	5.78 ± 1.20	0.001 *
Intermenstrual bleeding	7.55%	21.36%	16.67%	11.76%	19.42%	0.248 ^§^
Painful symptoms (VAS score)						
Dysmenorrhea	8.38 ± 1.12	7.61 ± 1.45	6.13 ± 3.29	5.64 ± 4.03	6.43 ± 3.18	0.487 *
Deep dyspareunia	5.39 ± 0.98	3.88 ± 2.15	1.69 ± 2.40	0.47 ± 1.32	1.13 ± 2.27	0.395 *
Dyschezia	5.66 ± 2.32	3.86 ± 2.66	1.25 ± 2.15	0	1.54 ± 2.82	0.135 *
Dysuria	8 ± 0	6.67 ± 2.42	0	0	0.50 ± 1.85	0.285 *
Painful symptoms (nr %)						
Dysmenorrhea	79.25%	77.67%	83.33%	70.59%	83.50%	0.671 ^§^
Deep dyspareunia	32.08%	17.48%	44.44%	11.76%	21.36%	0.006 ^§^
Dyschezia	26.42%	28.16%	36.11%	0	27.18%	0.095 ^§^
Dysuria	1.89%	5.83%	0.00%	0	7.77%	0.209 ^§^
Menstrual bleeding						
Menstrual bleeding (PBAC score)	62 ± 30.68	57.54 ± 20.85	58.83 ± 21.96	38.00 ± 8.59	62.05 ± 25.71	<0.00001 *
Heavy menstrual bleeding (PBAC ≥ 100)	18.87%	2.91%	5.56%	0	10.68%	0.006 ^§^

## Data Availability

The original contributions presented in this study are included in the article. Further inquiries can be directed to the corresponding author.
